# Unconscious processing of prototype heuristics in scientific innovation problem-solving

**DOI:** 10.3389/fpsyg.2023.1056045

**Published:** 2023-02-23

**Authors:** Yushi Ling, Liu Tan, Liyi Zhang, Guikang Cao

**Affiliations:** ^1^Faculty of Psychology, Southwest University, Chongqing, China; ^2^The High School Attached to Hunan Normal University-Meixihu High School, Changsha, China; ^3^Institute of Marxism, Chongqing Medical and Pharmaceutical College, Chongqing, China

**Keywords:** levels of consciousness, creative problem-solving, prototype heuristics, distractor tasks, scientific invention, insight, executive function

## Abstract

Previously published studies on the effect of how different levels of unconsciousness (UC) and different loads of executive functions (EFs) affect insight problem solving are inconsistent. In a set of three experiments, we used scientific innovation problems (SIP) as insight metrics and distractor tasks to induce UC. Experiment 1 confirmed that, compared with conscious processing, unconscious processing is more conducive to obtaining prototype heuristics for correctly solving scientific innovation problems creatively. Furthermore, Experiment 2 found that different levels of unconscious processing, which were induced by different distractor tasks, made a different impact on high or low difficulty creative problem solving. Experiment 3 indicated that unconscious processing could improve prototype activation and the ability to use key heuristics information in prototype heuristics processing by improving working memory, inhibitory control, and shifting ability of EFs. Overall, the present results provide additional evidence for the role of consciousness levels in insight problem solving.

## 1. Introduction

### 1.1. The prototype heuristics theory in the scientific invention problems

Creativity is the ability to produce both novel and appropriate work (Sternberg and Lubart, 1991, 1996), and insight is an important topic in the research of creativity in psychology (Dietrich and Kanso, 2010; Tong et al., 2013). Sometimes, a flash of inspiration or intuition may trigger a critical moment of thought that leads to an “aha” moment and solves a problem, known as insight. For example, the well-known golden crown problem. That is, Archimedes, the famous Ancient Greek philosopher, was asked to estimate whether the golden crown was made from pure gold. He was very confused at the beginning, but a solution suddenly hit him during his bath time because he found that when he got into the bathtub, once the water has been drained from the bathtub, objects of the same weight and density should drain the same volume of water. He realized that this discovery could solve the golden crown problem. This anecdote is a good example of insight (Hao et al., 2013; Zhu et al., 2019).

To further illustrate, we can treat the phenomenon that the object drains water out of a bathtub as a prototype, realize the connection between that prototype and the golden crown problem, and then apply that prototype to that problem; this is a way to solve the insight problem, that is, the prototype heuristic (Zhang et al., 2004). During the prototype heuristic process, the activation of a semantic representation of a prototype that benefits insight problem-solving is known as prototype activation, and the application of the heuristic information implied by the prototype (such as principles, rules, and methods) leads to successfully solving the insight problem of creativity (Zhang et al., 2012).

Many recorded insights, such as the golden crown problem, were derived from scientists and inventors (Ovington et al., 2018), which may suggest that a large part of real-world epiphanies come from scientific inventions. Most published studies that concentrate on the cognitive mechanism of insight more frequently adopt the compound remote associate problem (RAT; Beaty et al., 2014) and the puzzle task (Wu, 2007), and they all have a common problem, that is, artificial materials and lack of ecological validity (Tong et al., 2013, 2015; Yang et al., 2016). To solve this problem, Zhu (2011) used scientific innovation problems (SIPs) as experimental materials and compiled the Scientific Innovation Problems Database. Unlike divergent thinking, convergent thinking, or analogical transfer problems, SIPs comprise knowledge-rich problems (Yang et al., 2022). In this experimental material, each SIP includes contextual information of the scientific innovation problem, a prototype associated with it, and a reference answer. For example, groundwater needs to be pumped to irrigate crops in arid areas, but it uses too much electricity and is expensive to drill wells. This makes it difficult to scale up in the vast arid areas of the west (contextual information). The question is how to use groundwater without electricity or drilling wells. The relevant prototype information is that trees use capillary action in their roots to transport underground water from their roots to leaves hundreds of feet above the ground. Researchers always combine the SIP with the “learning-testing” paradigm to explore the effect of the prototype heuristic. Participants need to learn the prototype information first; the specific operation is to present one or more prototypes to the participants for learning without limitation of time, and after the participants report the completion of learning, researchers present them with target problems to explore whether the participants could activate the previously learned prototype to help solve the current creative problem (Yan et al., 2011). This is known as the prototype heuristic paradigm, which considers knowledge-rich contexts and enables more ecologically valid investigations of creative problem-solving in the laboratory (Yang et al., 2022). Moreover, the Scientific Innovation Problems Database has also been widely used in research (Zhu et al., 2017, 2019).

### 1.2. The influence of levels of consciousness on prototype heuristic and insight

Dijksterhuis and Meurs (2006) suggest that, compared with conscious thinking, unconscious thinking is more “liberal” and leads to “less obvious, less accessible, and more creative” ideas. Compared with non-insight problem-solving, insight problem-solving relies more on implicit, bottom-up, unconscious processes (Lebed and Korovkin, 2017; Stuyck et al., 2022). Simultaneously, the processing of the prototype heuristic includes unconscious thinking as well. For example, Cao et al. (2006) found that prototype activation has no difference between participants who implicitly or explicitly learned the prototype. Furthermore, they suggested that prototype activation could occur unconsciously and does not need conscious induction and summary. Hereafter, the process of matching various information of the prototype with the problem to find the solution to the problem is completed through conscious processing. A recent study by Xing et al. (2018) also showed that heuristics from prototypes probably involve an implicit, unconscious process.

Zhao (2018) identified that conscious and unconscious processing are both involved in the creative problem-solving process, meanwhile, Zhao also verified that the level of UC has deep and shallow processing by the sandwich masking and distractor task paradigms. The sandwich masking paradigm reduces or disappears the visibility of the target stimulus through the continuous and rapid presentation of the two stimuli, thus achieving unconscious-level processing. The distractor task is a task that causes the unconscious level processing of the target task through a task that occupies more cognitive resources. Sio and Ormerod (2009) used meta-analysis to assess the incubation effect of RAT tasks; their results also show that, compared with those difficult distractor tasks that consume more cognitive resources, the remote association of target words was observed faster in the easy distractor tasks with less cognitive resources. Overall, conscious and unconscious processing is involved in creative problem-solving, and different levels of UC will have different effects on creative problem-solving. Moreover, it could be argued that different levels of UC will also have different effects on prototype heuristics.

### 1.3. Executive functions

As mentioned earlier, conscious thinking, which involves bottom-up processing, and insight problem-solving appear to be closely linked. However, EFs, which involve top-down processing, do not rely on instinct or intuition (Diamond, 2013). Furthermore, an important factor of insight problem-solving, such as progress monitoring theory (Knoblich et al., 1999) developed by modern cognitive psychology, is that effective insight problem-solving involves substantial loads on working memory (an EF process). Chrysikou (2019) further points out that the importance of cognitive control mechanisms for creative thinking is a consensus in the field of creative neuroscience.

EFs refer to a series of higher cognitive abilities of individual consciousness and effective control of thinking and behavior, which include inhibition, shifting, and updating (Miyake et al., 2000). Inhibition is the repression of automatic reactions in the cognitive process or content, which mainly prevents irrelevant information from entering and being stored in working memory. Shifting means individuals respond to new situations with appropriate reactions and maintain cognitive and behavioral flexibility. That is, when faced with multiple tasks competing for a cognitive resource, the control process of attentional switching in these tasks takes precedence. Updating is the process by which an individual continuously incorporates new information and discards irrelevant information to the current task to change the contents of working memory based on the information presented. These three sub-functions are related to each other, but they play different roles in complex cognitive processes and play an important role in insight problem-solving.

For example, four fluid reasoning tests, 13 working memory tasks, and an intensive range of insight tasks were used by Chuderski and Jastrzebski (2018) to verify the relationships among the three; they found a strong positive correlation between EFs and insight problem-solving of 0.795, which verified a strong link between the two of them. Moreover, they also found that the working memory capacity factor explained 51.8% of insight variance, as well as 87.0% of reasoning variance. Xing et al. (2018) also found positive correlations between EFs and insight problem-solving, and updating (an EF) significantly predicted insight performance. Cassotti et al. (2016) suggested that inhibitory control is a central process in creative problem-solving and idea generation from childhood through adulthood because developing a solution to a creative problem requires suppressing inappropriate thoughts. In addition, EEG research by Benedek et al. (2012) showed that inhibitory control resources were positively correlated with creative task scores (Benedek et al., 2012; Beaty et al., 2014).

To summarize, an antagonistic relationship between UC and EFs may exist; however, unconsciousness, EFs, and its three components play a role in promoting the performance of insight problem-solving. So, what are the true relationships among UC, EFs, and insight problem-solving? Previous research has established some models to explore the relationships among these three constructs. For example, the associative theory contends that UC promotes insight problem-solving (Mednick, 1962); however, this theory does not consider the role of EFs. Recent research by Stuyck et al. (2022) claimed that they proved the associative theory as they found that cognitive control did not influence the performance of insight problem-solving. In their research, the performance of insight problem-solving was measured by RAT grades with an accuracy of 91%–94%. Of note, however, previous research has already provided evidence that when RAT difficulty was very high (all but one of 39 participants were able to solve no more than one problem out of nine), the promoting effect of unconscious processing on RAT performance could be observed, but when RAT difficulty was medium (correct answer rates were between 41% and 59% with 39 participants), the promoting effect disappeared (Zhong et al., 2008). The results of Zhong et al. (2008) may reveal that the research by Stuyck et al. (2022) is insufficient to conclude that cognitive control did not influence the performance of insight problem-solving. In addition to the association theory, some researchers claimed that only EFs could promote insight problem-solving, such as Chuderski and Jastrzebski (2018) who attributed their results to working memory playing a central role in insight problem-solving and “nothing special with special add-ons.” Although this view considers the role of EFs, it ignores the UC that already exists. Early in 2007, Schmeichel (2007) found that working memory tasks could deplete EFs. Therefore, the working memory task can also play the role of a distractor task, making the insight problem-solving processing into the unconscious thinking state. Thus, it is inappropriate to consider only the role of working memory. The view of associative processes and executive control both playing a role in insight problem-solving has also been proposed. To illustrate, Beaty et al. (2016) argued that the default network influences the generation of candidate ideas, but the control network can constrain and guide the process through top-down monitoring and executive control to meet the goals of a particular task. However, they also make it clear that the framework does not include cases of creative insight. Therefore, how EFs and UC play a role in insight problem-solving remains to be explored.

According to Beaty et al. (2016), in creative problem-solving, the default network influences the generation of candidate ideas before the constraint and guidance of executive control. Therefore, we believe that only after the UC induces the generation of ideas, will EFs play a role. Otherwise, the process of logical reasoning or functional fixation can only occur. On the other hand, if the EFs are not functioning, individuals may not be able to report correct opinions even with unconscious thinking. Therefore, we hypothesize that EFs mediate the relationship between UC and insight problem-solving.

### 1.4. Current research

To sum up, in solving creative problems, sometimes unconscious processing may be better than conscious processing results. In addition, the effect of unconscious processing induced by a low cognitive load distractor task (low-distractor task) on creative problem-solving is greater than that of a high cognitive load distractor task (high-distractor task). Research has also shown that improvements in certain cognitive abilities in EF can also boost creative performance. However, although sufficient research exists on the relationships among UC, EFs, and insight problem-solving, no clear explanation has been determined. Therefore, we report three experiments to explore the relationships between UC and EFs as well as how they play a role in insight problem-solving.

To this end, the purpose of Experiment 1 was to verify whether unconscious processing is better than conscious processing in SIP solving and whether item difficulty will influence performance. Most researchers investigating UC have utilized the distractor task (Ding et al., 2019), mask priming paradigm (Huber-Huber and Ansorge, 2018; Silva et al., 2018), and dual-task paradigm (Lebed and Korovkin, 2017). In Experiment 1, a distractor task was used wherein participants were required to perform several comparison tasks in the process of problem-solving. Such concurrent cognitive control tasks would prevent cognitive control from playing a role in the main task (Huber-Huber and Ansorge, 2018); thus inducing the participants to engage in unconscious thinking. Based on the empirical results mentioned earlier, we predicted that participants who performed the distractor task performed better on insight problem-solving than participants who performed conscious thinking, especially when the problem was difficult.

Experiment 2 added different levels of consciousness. We speculate that the more difficult the distractor task, the more cognitive resources will be occupied, and the higher the level of UC. According to previous studies, too much occupation of cognitive resources will lead to poorer performance in SIP solving than less occupation.

A measure of EFs was introduced in Experiment 3 to further explain the results of Experiment 2. EFs are a higher-level cognitive ability used in careful research and goal realization (Cristofori et al., 2019). As an ability, researchers assume that it will not change in the short term. Therefore, many researchers have focused their attention on individual differences in EFs (Steward et al., 2018; Stolte et al., 2020; Tsai et al., 2021). However, Schmeichel (2007) believed that, similar to the ego depletion hypothesis (Baumeister et al., 1998), EFs have a depletable capacity. Their study found that, compared with the control group, participants who completed the distractor task showed decreased performance in the next EFs measuring. Moreover, participants who previously completed the inhibition task had a negative influence on the subsequent working memory updating task and vice versa (the previous working memory updating task had a negative impact on inhibition task performance). This suggests that the completion of tasks involving EFs impacts subsequent measures of EFs. Specifically, the previous task consumes a portion of the limited resources of EFs, thus reducing the amount of EFs available later. To test this idea, EFs were measured immediately after the distractor task in Experiment 3 to explore whether the distractor task affected EFs and whether the affected EFs would lead to different SIP-solving results. In addition, although the EFs measurement task itself also occupied cognitive resources, the three groups of subjects completed the same EFs measurement after the level of consciousness manipulation; therefore, theoretically, the occupied cognitive resources are equal and can be balanced.

## 2. Experiment 1

Given the evidence that UC has been effective in creativity, especially in difficulty problem-solving, we verified whether, when SIP is used to test creativity, different levels of consciousness have different effects on it. We hypothesize that when the SIP is difficult, UC can promote prototype heuristics in solving problems more than consciousness.

### 2.1. Method

#### 2.1.1. Participants

Seventy-eight participants (aged between 18 and 27 years, mean age = 21.48 years, SD = 1.68) from Southwest University were recruited. Participants were randomly assigned to the conscious condition (*n* = 38) and the unconscious condition (*n* = 40). After removing one subject who failed to complete all the experimental tasks, the final number of effective subjects was 77. All participants provided informed consent before participating and received some remuneration after the experiment. Experimental protocols for all three experiments were approved by the University’s local ethics committee.

#### 2.1.2. Materials

##### 2.1.2.1. Scientific innovation problem

We chose 84 SIPs from the Scientific Innovation Problems Database (Zhu, 2011) based on difficulty. Three students with psychology as a major were asked to rate the difficulty of the questions on a 7-point scale (1 = lowest difficulty and 7 = highest difficulty). The order of each question was presented randomly. The scorer reliability was 0.89. According to the scoring results, 20 questions were selected for the high-difficulty condition (MD = 4.70, SD = 0.52) and 20 questions for the low-difficulty condition (MD = 2.10, SD = 0.48).

##### 2.1.2.2. Distractor task

Several comparison tasks were adopted to induce UC. A random set of numbers appeared on both the left and right sides of the computer screen. The numbers were random integers between 10 and 99 and appeared for only 1 s. The participants were asked to quickly and accurately determine which side of the screen had a larger number and to respond accordingly. If the number on the left side of the screen was larger, the participant pressed “Q,” and if the number on the right side of the screen was larger, the participant pressed “P.” Participants were asked to perform a 3-min distractor task. This is because previous studies have found that when the distractor task lasts 3 min, it has the best effect on creativity compared to 1 or 5 min (Gilhooly, 2016).

#### 2.1.3. Procedure

The experiment was programmed using E-Prime 2.0 and consisted of four phases: problems presentation, prototypes learning, consciousness level manipulation, and answer.

In the problems presentation phase, the participants were presented with eight blocks. Each block had five trials that would randomly present a SIP on the computer screen (high-and low-difficulty problems were presented randomly too), resulting in 40 trials presenting 40 problems. The participants had 30 s to memorize it carefully. To eliminate participants answering questions based on personal experience, instead of learning from the prototype we provided, we also required the participants to judge whether they already knew the answer to the question due to personal life experience or education. Participants were asked to press the “F” key if they already knew the answer before participating in this study and the “J” key if not. The answer was eliminated in the data analysis phase if participants pressed the “F” key.

The prototypes learning phase used the same design as the problems presentation phase, but each trial was randomly presented with the prototype information corresponding to the problem in the first phase (but the problem itself was not presented), such as “When the nurse gives the injection, she can use a small needle to inject the medicine like squeezing toothpaste,” and participants did not have to press any key, then after 60 s, the screen will automatically display the next prototype until all prototypes corresponding to the problem are presented.

In the consciousness level manipulation phase, participants in the unconscious group were informed to complete a 3-min number comparison task; participants in the conscious group were instructed to recall the problems and prototypes they had seen before and to try to think of solutions to the problems.

In the answer phase, the participants began to answer the SIP with paper and pen.

#### 2.1.4. Data analysis

For the SIP, we rated the answers on a scale from 0 to 2, based on the criteria shown in previous studies (Yang et al., 2016). If the participant had recalled the correct prototype and correctly solved the problem, the score was 2; if the participant had only recalled the correct prototype but failed to solve the problem, the score was 1; and if the participant had failed to answer the question correctly, the score was 0. To assess how well the participants solved the problem, we computed two indices, one was the prototype activation rate, which refers to the number of questions with a non-zero individual score divided by the number of all questions after eliminating the questions with known answers. The other was the accuracy rate, which refers to the number of questions with an individual score of 2 divided by the number of all questions, after excluding questions to which the participant knew the answer. Hereafter, SPSS 22.0 was used for statistical analysis. Repeated measures ANOVA was performed for the prototype activation rate and problem-solving accuracy rate.

### 2.2. Results

Three trained psychology majors were asked to rate participants’ SIP-solving scores according to the method we presented in the Data Analysis section earlier. The scorer reliability was 0.918. Descriptive statistics for the prototype activation rate and accuracy rate are provided in Table 1.

**Table 1:** Descriptive statistics of prototype activation rate and accuracy rate in conscious and unconscious processing conditions (M ± SD).

	Conscious		Unconscious	
	High difficulty	Low difficulty	High difficulty	Low difficulty
Prototype activation rate	0.765 (0.11)	0.893 (0.09)	0.918 (0.06)	0.922 (0.06)
Accuracy rate	0.313 (0.12)	0.598 (0.17)	0.668 (0.13)	0.718 (0.11)

#### 2.2.1. The prototype activation rate

A 2 × 2 repeated-measures ANOVA was performed to assess the effects of the within-subjects factor difficulty (high difficulty vs. low difficulty) and the between-subjects factor group (conscious vs. unconscious) on the prototype activation rate; age and gender of participants were used as covariables. A significant main effect of the group [*F*_(1,76)_ = 32.732, *p* < 0.001, *η_p_^2^* = 0.304] revealed that the prototype activation rate of the conscious condition (M = 0.827, SD = 0.085) was significantly lower than the unconscious condition (M = 0.920, SD = 0.049). The main effect on difficulty was also significant [*F*_(1,76)_ = 46.172, *p* < 0.01, *η_p_^2^* = 0.381]; the high-difficulty prototype activation rate (M = 0.840, SD = 0.116) was significantly lower than the low-difficulty prototype activation rate (M = 0.907, SD = 0.077). Moreover, the interaction between group and difficulty was significant, *F*_(1,76)_ = 40.509, *p* < 0.001, *η_p_^2^* = 0.351.

**Figure 1:**
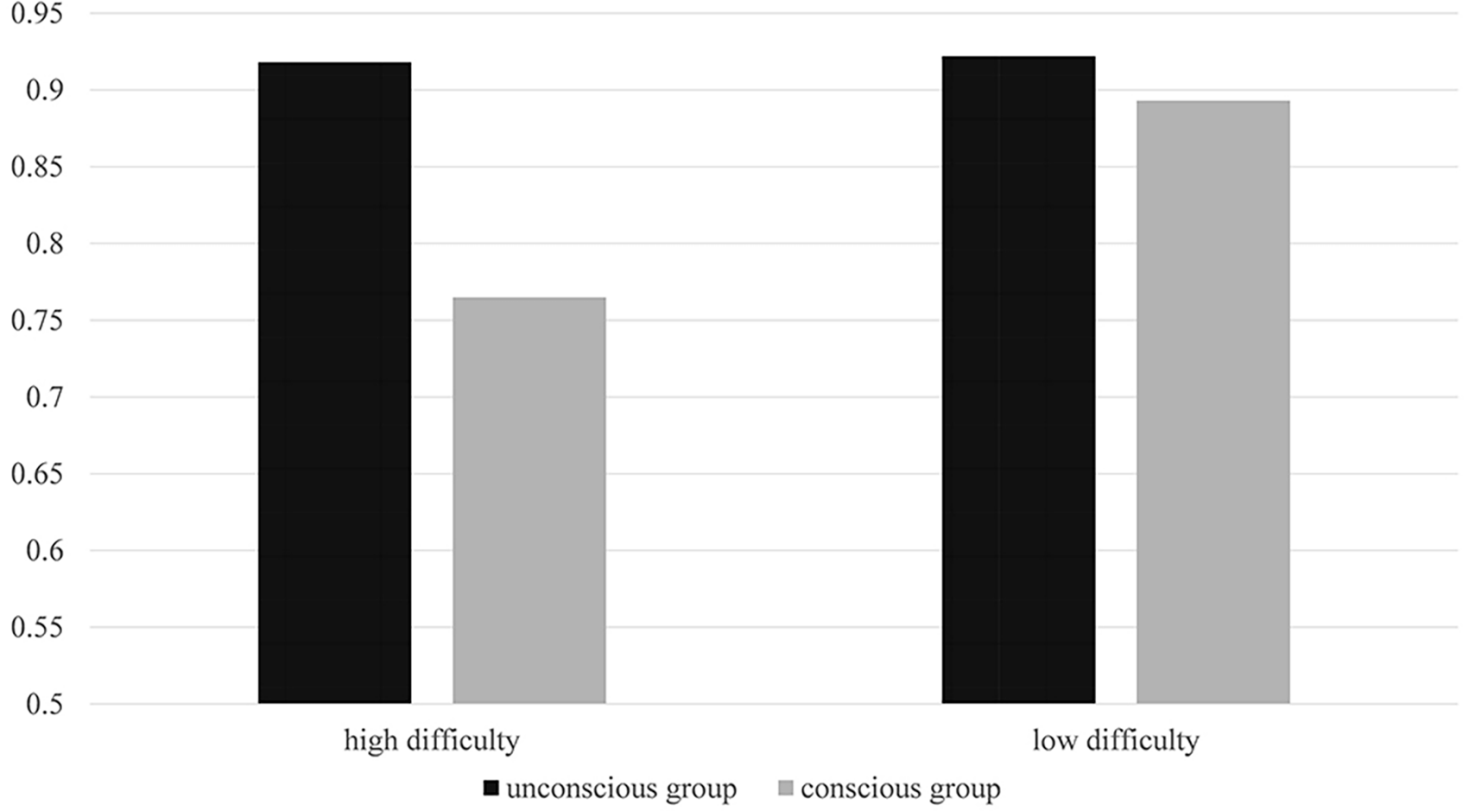
The prototype activation rate of Experiment 1.

As we found a significant interaction between group and difficulty, we followed up with a simple effect analysis. The outcomes showed that the prototype activation rate was not significantly different (*p* = 0.095) between the unconscious (M = 0.922, SD = 0.055) and conscious groups (M = 0.893, SD = 0.092) under the low difficulty condition. However, in the high-difficulty condition, the prototype activation rate of the unconscious group (M = 0.918, SD = 0.055) was significantly higher than the conscious group (M = 0.765, SD = 0.110; *p* < 0.01). In the conscious condition, the prototype activation rate of the high-difficulty task (M = 0.765, SD = 0.110) was significantly lower than the low-difficulty task (M = 0.893, SD = 0.092). However, there was no difference between the high-difficulty prototype activation (M = 0.918, SD = 0.055) and low-difficulty prototype activation rates (M = 0.922, SD = 0.055) in the unconscious group. The results are shown in Figure 1.

#### 2.2.2. The accuracy rate

A 2 × 2 repeated measures ANOVA was performed with the accuracy rate as the dependent variable, difficulty (high vs. low difficulty) as the within-subject variable, group (conscious vs. unconscious) as the between-subjects variable, and age and gender as the covariables. The results showed that the main effect was highly significant, *F*_(1,76)_ = 71.199, *p* < 0.001, *η_p_^2^* = 0.487. The accuracy rate of the conscious condition (M = 0.455, SD = 0.138) was significantly lower than the unconscious condition (M = 0.693, SD = 0.106). The main effect on difficulty was significant, *F*_(1,76)_ = 193.755, *p* < 0.001, *η_p_^2^* = 0.721; the high difficulty accuracy rate (M = 0.488, SD = 0.216) was significantly lower than the low difficulty accuracy rate (M = 0.657, SD = 0.156). The interaction between group and difficulty was significant, *F*_(1,76)_ = 94.566, *p* < 0.001, *η_p_^2^* = 0.558.

**Figure 2:**
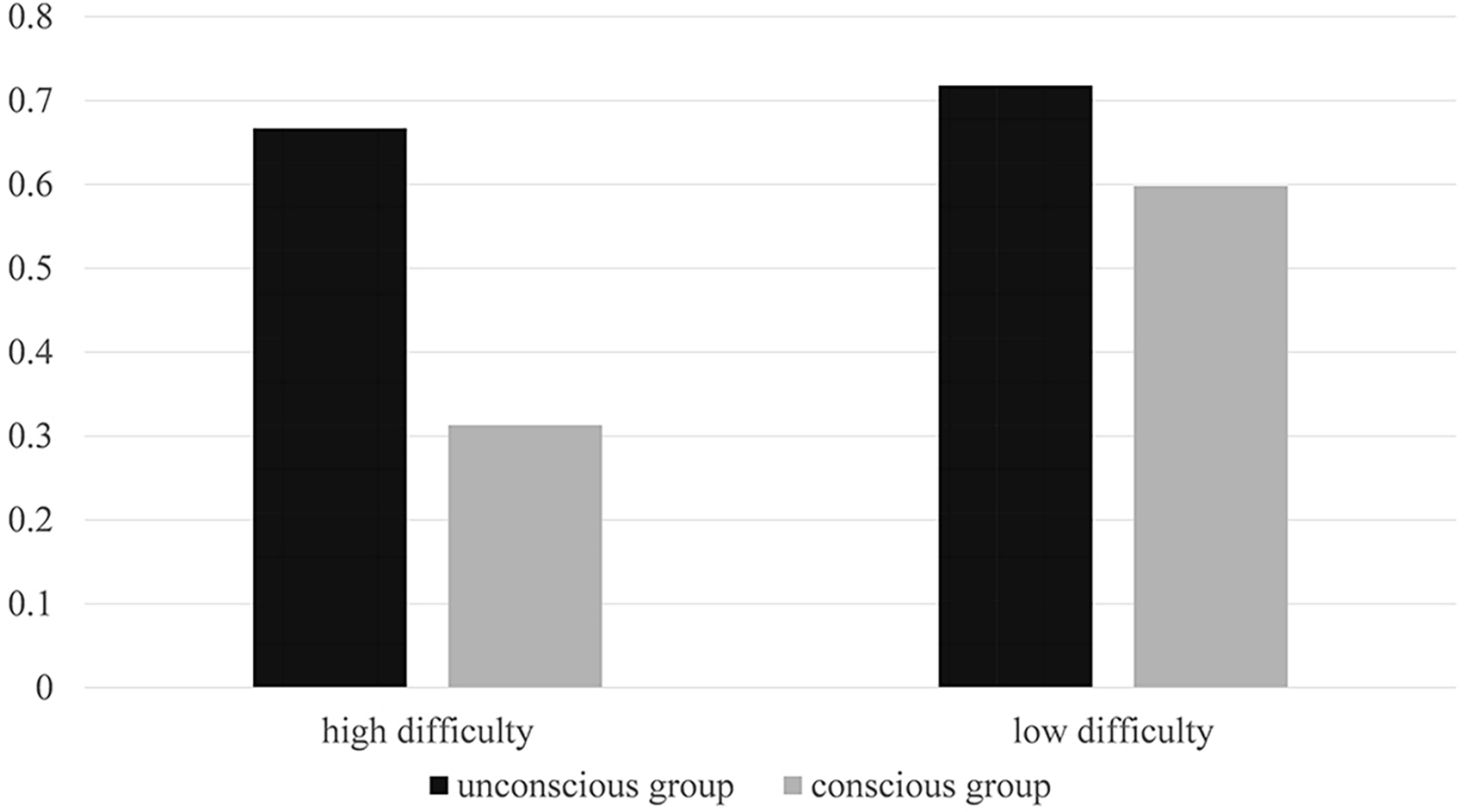
The accuracy rate of Experiment 1.

Further simple effect analysis showed that the accuracy rate of the unconscious group (M = 0.718, SD = 0.110) was significantly higher than the conscious group (M = 0.598, SD = 0.172; *p* < 0.01) under the low-difficulty condition, and the accuracy rate of the unconscious group (M = 0.667, SD = 0.125) was significantly higher than the conscious group (M = 0.313, SD = 0.121; *p* < 0.01) under the high-difficulty condition. In the unconscious processing group, the accuracy rate of the high-difficulty task (M = 0.668, SD = 0.125) was significantly lower than the low-difficulty task (M = 0.598, SD = 0.172; *p* = 0.004). Moreover, there was no difference between the high-difficulty (M = 0.667, SD = 0.125) and low-difficulty accuracy rates (M = 0.598, SD = 0.172). The results are shown in Figure 2.

### 2.3. Discussion

Taken together, these results showed that if we solve SIP consciously, two indices of prototype heuristics (the prototype activation and accuracy rates) can be quite different because of the different task difficulties. Otherwise, the effect of prototype heuristics is good regardless of the difficulty level of the task if unconscious processing is used. When the task difficulty was low, the prototype activation rate of the unconscious group was no different from that of the conscious group, but the accuracy rate was significantly higher than that of the conscious group. When the task difficulty was high, the prototype activation rate and accuracy rate of the unconscious group were significantly higher than that of the conscious group. This result is consistent with our hypothesis, that is, UC promotes SIP solving. According to previous research, the prototype heuristic is an automatic process (Zhu et al., 2019), specifically, there is a semantic similarity between the “need function” in problem representation and the “characteristic function” in prototype representation. When participants map the “characteristic function” to the “need function,” the problem will be solved, and such structural mapping is an automatic process (Zhang et al., 2012), also known as representation-connection (Zhu et al., 2019). In SIP solving, individuals need to find the prototype character that plays a key role in the current problem among the numerous prototype characters, which requires a wide range of information processing. Unconscious processing, with its powerful searching and associative abilities, can help individuals find corresponding archetypes and solve problems.

Both simple and difficult scientific inventions benefited from unconscious processing, which is somewhat different from previous studies. Zhong et al. (2008), using RAT as a creative task, showed that when the difficulty of the task was simple, there was no significant difference in the impact of conscious and unconscious thought on creative problem-solving, but when the difficulty of the task was medium, unconscious thought had a more prominent role in promoting creative problem-solving. In our study, UC was significantly more conducive to creative problem-solving than consciousness, regardless of whether the task was easy. This occurred presumably because different creative tasks were used. From the perspective of semantic processing, RAT requires the semantic processing of words, while SIP requires a semantic connection between sentences. It may even be that the low-difficulty SIP is more difficult than the RAT task, so it is more suitable for unconscious processing. Due to different creative tasks, the definition of difficulty may also be different. To illustrate, the difficulty of the materials in this experiment was subjectively assessed by three students majoring in psychology, while in the Scientific Innovation Problem Database, each question has a corresponding heuristic index. A heuristic index refers to the accuracy rate of solving problems obtained by the participant after learning the prototype minus the accuracy rate of solving problems without learning the prototype. Therefore, in Experiment 2, the heuristic index was used as the difficulty standard of SIP to further explore whether the facilitation of unconscious processing in creative problem-solving was related to difficulty.

Overall, the results provide strong support that distractor tasks can promote problem-solving after leading to the individual unconscious thought. It is worth further discussing that if the distractor task occupies too many cognitive resources, will the promotion effect of UC on creative problem-solving be weakened? To address this, we conducted Experiment 2.

## 3. Experiment 2

The results of Experiment 1 suggested that UC has a facilitatory effect on prototype heuristics. Will this phenomenon be affected by different cognitive loads induced by different difficulties of distractor tasks? We hypothesized that UC’s positive effect on prototype heuristics would be reduced when the distractor task becomes more difficult and consumes more cognitive resources.

### 3.1. Method

#### 3.1.1. Participants

Ninety participants (aged between 18 and 23 years, mean age = 19.67 years, SD = 1.29, 36 male participants) from Southwest University were recruited by advertising. Participants were randomly assigned to the conscious condition (*n* = 30), low-distractor task condition (*n* = 30), and high-distractor task condition (*n* = 30). All participants provided informed consent before participating and received some remuneration after the experiment.

#### 3.1.2. Materials

##### 3.1.2.1. Scientific innovation problem

Twenty-four SIPs were selected from the Scientific Innovation Problems Database (Zhu, 2011). Twelve of them were low difficulty (M = 0.81, SD = 0.05) and the others were high difficulty (M = 0.57, SD = 0.02). Importantly, we measured difficulty by heuristics rate.

##### 3.1.2.2. Distractor task

Experiment 2 adopted the same distractor task as Experiment 1; the only difference was that different numeric types were used to induce high and low cognitive loads. As comparisons between fractions are more complicated compared with integer comparisons, fraction comparisons require a higher cognitive load to process. Therefore, we induced low cognitive loads by requiring participants to compare random two-digit numbers and induced high cognitive loads by requiring participants to make comparisons between random fractions, in which the numerator and denominator were both random integers between 10 and 99. A preliminary experiment was used to examine the cognitive load distinction between integer and fraction comparisons. As a result, the accuracy of fraction comparison tasks (M = 0.73, SD = 0.89) was significantly lower than the accuracy of integer comparison tasks (M = 0.92, SD = 0.45), reaction time (M = 928.79 ms, SD = 205.03) was significantly higher than that of integer comparison tasks (M = 613.77 ms, SD = 135.93), and the difficulty score (M = 5.90, SD = 0.89), which was subjectively assessed by participants, was significantly lower than the difficulty score of integer comparison tasks (M = 1.33, SD = 0.69). It could be argued that the integer and fraction comparison tasks have a reliable effect on distinguishing between low and high cognitive loads.

#### 3.1.3. Procedure

The basic procedure in Experiment 2 was identical to Experiment 1, with two alterations. One was that in the problems presentation phase, the participants were presented with four blocks instead of eight, and each block had six trials that would randomly present a SIP on the computer screen, resulting in 24 trials presenting 24 problems. The other alteration was that in the consciousness level manipulation phase, participants in the low-distractor task condition were informed to complete a 3-min integer comparison task, participants in the h-distractor task condition were informed to complete a 3-min fraction comparison task, and the conscious condition participants were informed to recall the problems and prototypes they had seen before and to try to think of solutions to the problems.

### 3.2. Results

SPSS 22.0 was used for statistical analysis. Repeated measures ANOVA was performed for the prototype activation and problem-solving accuracy rates. Three trained psychology majors were asked to rate participants’ SIP-solving scores according to the method we presented in the Data Analysis section in Experiment 1. The scorer reliability was 0.848. Descriptive statistics for the prototype activation and the accuracy rates are provided in Table 2.

**Table 2:** Descriptive statistics of prototype activation rate and accuracy rate in different conditions (M ± SD).

	Conscious condition		Low cognition loads condition		High cognition loads condition	
	High difficulty	Low difficulty	High difficulty	Low difficulty	Low difficulty	High difficulty
Prototype activation rate	0.752 (0.158)	0.806 (0.119)	0.938 (0.076)	0.927 (0.066)	0.848 (0.086)	0.905 (0.075)
Accuracy rate	0.510 (0.138)	0.474 (0.175)	0.630 (0.808)	0.658 (0.118)	0.651 (0.102)	0.575 (0.127)

#### 3.2.1. The prototype activation rate

A 2 × 3 repeated-measures ANOVA was performed to assess the effects of the within-subjects factor difficulty (high difficulty vs. low difficulty) and the between-subjects factor group (conscious vs. low-distractor task vs. h-distractor task) on prototype activation rate; age and gender of participants were used as covariables. A significant main effect of the group [F_(2,85)_ = 26.552, *p* < 0.001, *η_p_^2^* = 0.358] revealed that the prototype activation rate of the low-distractor task condition (M = 0.933, SD = 0.050) was significantly higher than the high-distractor task condition (M = 0.876, SD = 0.061), and the prototype activation rate of the high-distractor task condition was significantly higher than that of the conscious condition (M = 0.778, SD = 0.125). The main effect on difficulty was not significant [*F*_(2,85)_ = 0.015, *p* = 0.903, *η_p_^2^* < 0.001]. The interaction between groups and difficulty was significant, *F*_(2,85)_ = 3.625, *p* = 0.031, *η_p_^2^* = 0.079.

**Figure 3:**
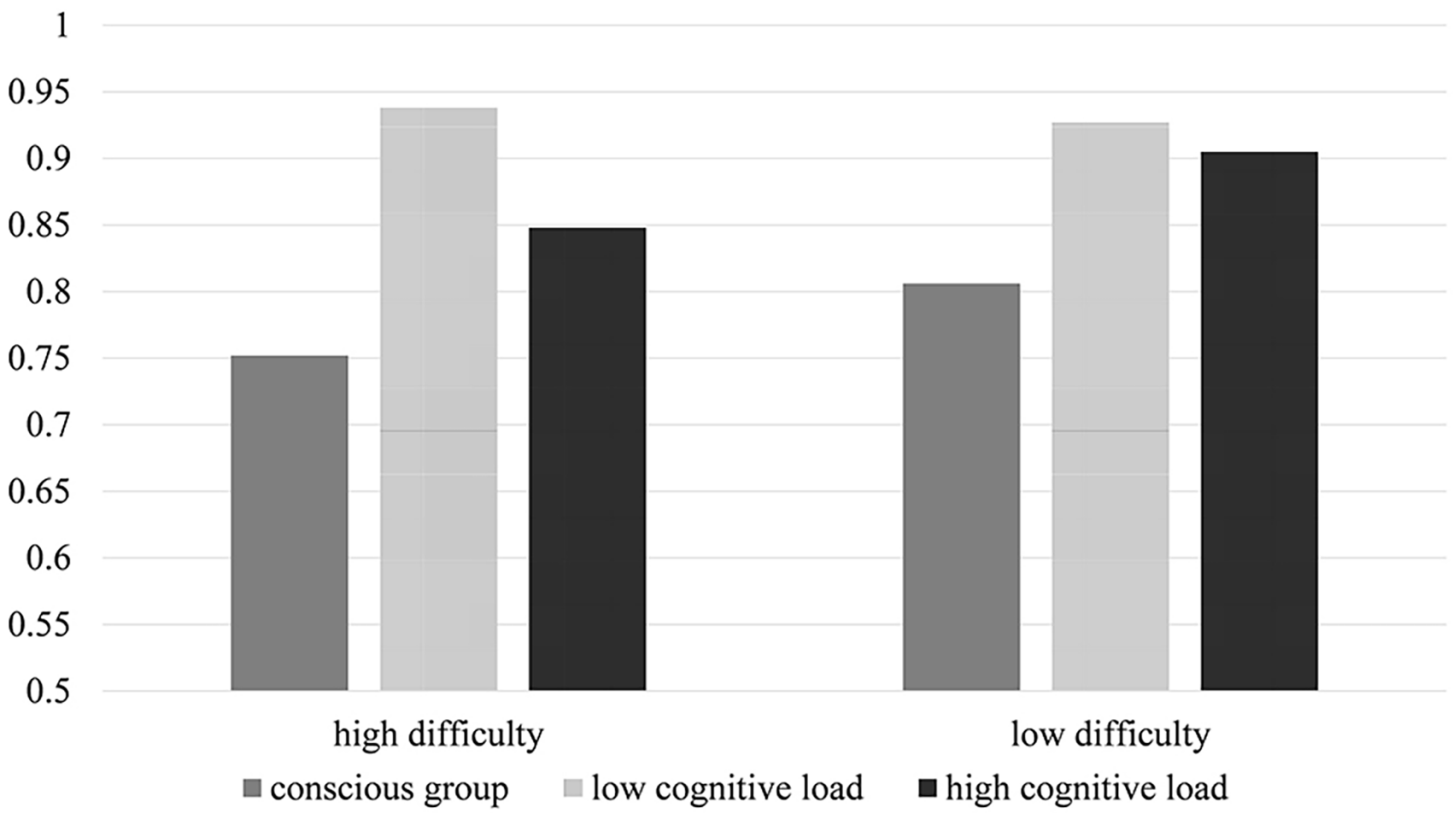
The prototype activation rate of Experiment 2.

Simple effect analysis showed that the outcomes indicated that the prototype activation rate in the conscious condition (M = 0.805, SD = 0.017) was significantly lower than the low-distractor task condition (M = 0.928, SD = 0.016) and high-distractor task condition (M = 0.905, SD = 0.016; *p* < 0.001). However, the prototype activation rate was not significantly different between the high-and low-distractor task conditions (*p* = 0.319). However, when the problems were highly difficult, the prototype activation rate in the conscious condition (M = 0.750, SD = 0.021) was significantly lower than in the low-distractor task condition (M = 0.939, SD = 0.020) and high-distractor task condition (M = 0.848, SD = 0.020; *p* < 0.001), and the prototype activation rate in the high-distractor task condition was significantly lower than the low-distractor task condition (*p* < 0.001). Simultaneously, in the conscious condition, the prototype activation rate in high-difficulty problems (M = 0.750, SD = 0.021) was significantly lower than that in low-difficulty problems (M = 0.805, SD = 0.017; *p* = 0.011). In the low-distractor task condition, the prototype activation rate in high-and low-difficulty problems was not significantly different (*p* = 0.582). In the high-distractor task condition, the prototype activation rate in high-difficulty problems (M = 0.848, SD = 0.020) was significantly lower than that in low-difficulty problems (M = 0.905, SD = 0.016; *p* = 0.006). A visual display is provided in Figure 3.

#### 3.2.2. The accuracy rate

A 2 × 3 repeated-measures ANOVA was performed to assess the effects of the within-subjects factor difficulty (high difficulty vs. low difficulty) and the between-subjects factor group (conscious vs. low-distractor task vs. high-distractor task) on the accuracy rate; age and gender of participants were used as covariables. A significant main effect of the group [*F*_(2,85)_ = 15.109, *p* < 0.001, *η_p_^2^* = 0.258] revealed that the accuracy rate of the low-distractor task (M = 0.644, SD = 0.080) and high-distractor task conditions (M = 0.613, SD = 0.098) were significantly higher than that of the conscious condition (M = 0.492, SD = 0.149), but the accuracy rate of the high-distractor task and low-distractor task conditions were not differentiated. The main effect on difficulty was significant [*F*_(2,85)_ = 5.217, *p* = 0.025, *η_p_^2^ =* 0.057]. Moreover, the interaction between groups and difficulty was significant, *F*_(2,85)_ = 6.092, *p* = 0.003, *η_p_^2^* = 0.123.

**Figure 4:**
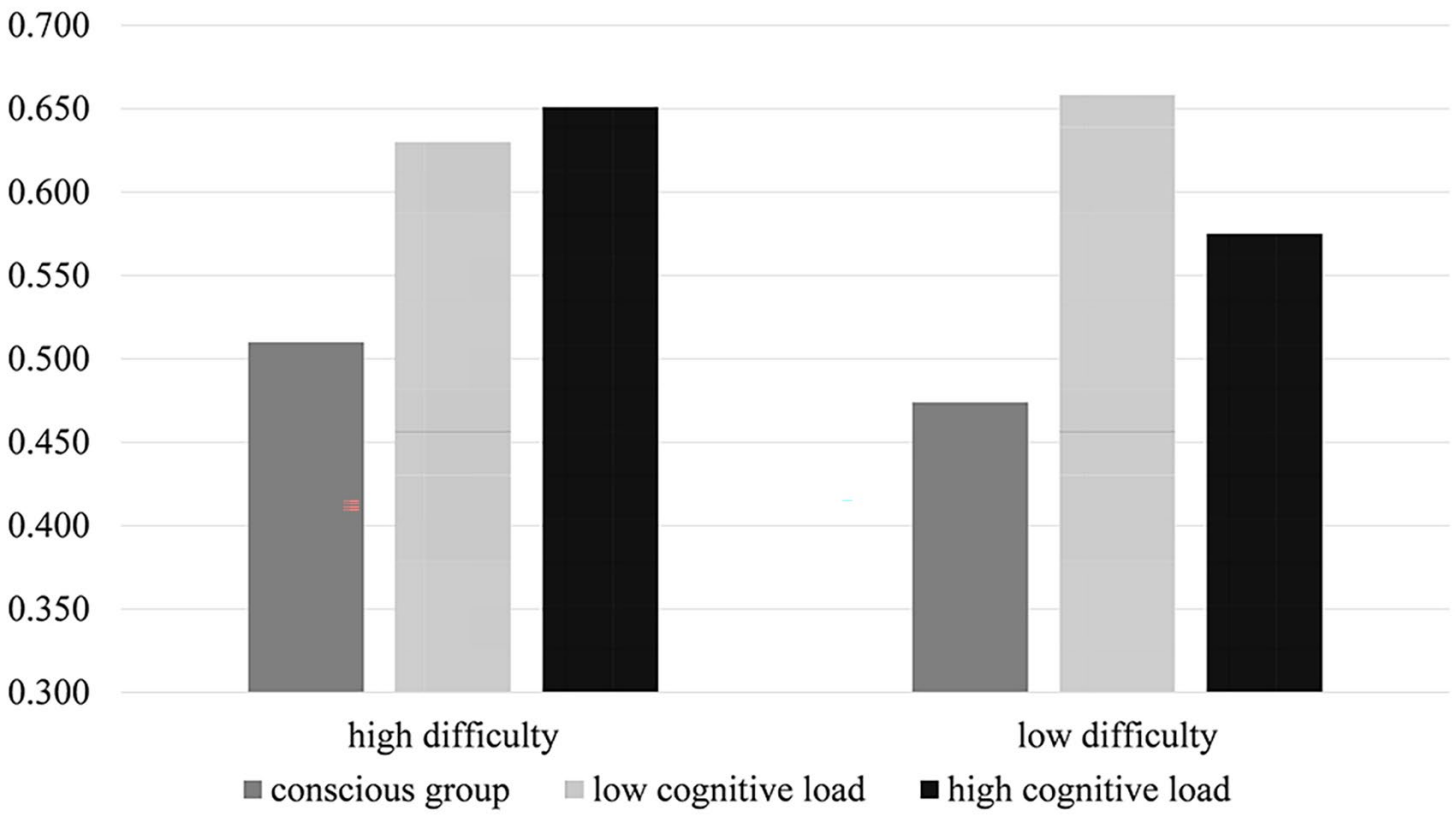
The accuracy rate of Experiment 2.

A simple effect analysis was conducted and the outcomes indicated that when SIPs were high-difficulty problems, the accuracy rate in the conscious condition (M = 0.510, SD = 0.138) was significantly lower than the low-distractor task (M = 0.630, SD = 0.808) and high-distractor task conditions (M = 0.651, SD = 0.102; *p* < 0.001), but the accuracy rate was not significantly different between the high-and low-distractor task conditions (*p* = 0.846). When SIP were low-difficulty problems, the accuracy rate in the conscious condition (M = 0.474, SD = 0.175) was significantly lower than the low-distractor task (M = 0.658, SD = 0.118; *p* < 0.001) and high-distractor task conditions (M = 0.575, SD = 0.127; *p* = 0.021), but the accuracy rate was not significantly different between the high-and low-distractor task conditions (*p* = 0.069). The accuracy rates of high-and low-difficulty problem-solving were not significantly different under the conscious and low-distractor task conditions (*p* = 0.10; *p* = 0.195), and a significant difference was found under the high-distractor task condition (*p* = 0.001). More specifically, the accuracy rate of high-difficulty problem-solving was significantly greater than that of low-difficulty problem-solving. A visual display is presented in Figure 4.

### 3.3. Discussion

In low-difficulty SIP solving, the prototype activation rate of the two unconscious groups was better than that of the conscious group, which partially supports the results of Experiment 1. Simultaneously, the prototype activation rate of the high-distractor task was significantly lower than that of the low-distractor task condition, which demonstrates that with an increase in cognitive load induced by the distractor task, the effect of unconscious thinking promoting creative problem-solving declined. This occurred presumably because although the participants were still unconsciously processing the SIPs when they were doing irrelevant tasks, the level of cognitive load of distractor tasks might affect the degree of involvement in the target and irrelevant tasks (Damian and Sherman, 2013). In prototype heuristics, to realize the connection between prototype information and SIPs, the individual needs to search out the corresponding information from all the currently learned prototypes to activate the prototype successfully. Previous research suggests that unconscious thinking made individuals conduct a wide range of searches, including information that may seem irrelevant to the problem (Ding et al., 2019). If the degree of involvement of the individual in the distractor task is too high, even though the activation of the prototype can be realized in unconscious processing, the individual may not be aware of it at the conscious level, thus reducing the individual’s activation rate in the prototype.

The accuracy rates were not significantly different between the three levels of consciousness. However, in the high-distractor task condition, the accuracy rate of high-difficulty problem-solving was significantly higher than the accuracy rate of low-difficulty problem-solving. These findings verified Zhong’s assumption, showing that conscious or unconscious thinking makes no difference in the promoting effect of problem-solving when the difficulty of problems is low; only high-difficulty problems could distinguish the promoting effect between conscious and unconscious thinking. According to Zhao (2018), unconscious processing could be divided into deep and shallow processing; deep processing could improve the accessibility of answers. Therefore, do the different levels of cognitive load induced by distractor tasks lead to changes in the depth of unconscious processing and further lead to differences in problem resolution rates? Furthermore, how does the depth of unconscious processing affect individual cognitive activities in problem-solving? To address this, we conducted Experiment 3.

## 4. Experiment 3

The results of Experiments 1 and 2 suggested that, compared with consciousness, UC had a facilitatory effect on prototype heuristics under high-difficulty problem-solving conditions, and the size of the facilitation effect is related to the cognitive load induced by the distractor task. This occurred presumably because UC induced by a distractor task changes individual EFs and influences creative problem-solving. Thus, we only chose high-difficulty SIPs to further investigate the internal mechanism of the promotion effect of unconscious processing on prototype heuristics. The experiments reported here try to verify whether unconscious thinking promotes individual EF, which is conducive to creative problem-solving.

We hypothesized the following: a. compared with conscious processing, unconscious processing occupied fewer EFs, and compared with low-distractor task, high-distractor task depleted more EFs; b. residual EFs in the unconscious state can promote SIP solving, and EFs play a mediating role between UC and SIP solving.

### 4.1. Method

#### 4.1.1. Participants

Eighty-six participants (aged between 18 and 23 years, mean age = 19.67 years, SD = 1.43, male = 31) from Southwest University were recruited by advertising. Participants were randomly assigned to the conscious condition (*n* = 28), low-distractor task condition (*n* = 29), and high cognitive loads condition (*n* = 29). All participants provided informed consent before participating and received some remuneration after the experiment.

#### 4.1.2. Materials

##### 4.1.2.1. Scientific innovation problem

Twenty-four high-difficulty (M = 0.63, SD = 0.05) SIPs were selected from the Scientific Innovation Problems Database (Zhu, 2011); and the difficulty was measured by heuristics rate.

##### 4.1.2.2. Distractor task

The distractor task in Experiment 3 was identical to Experiment 2.

##### 4.1.2.3. Executive functions measurement

The current study used a two-back task to examine the ability of individuals to update. During the task, random integer numbers between 0 and 9 were presented one at a time, and participants were asked to compare each number with the second number before it. If the two numbers are the same, a key response is required. If the two numbers are different, participants do not react. Each number was presented for only 1 s, requiring participants to react as quickly as possible. In data processing and analysis, we excluded the reaction time of the wrong reaction of the participants and then calculated all the reaction times of the correct trial.

The shifting number task examined the ability of individuals to shift. In each trial, a single letter and a single number were presented on the screen concurrently, the word color could be red or green, and stimuli would change color randomly. Participants responded to stimuli by pressing keys. If the word color was green, participants needed to respond to the parity of numbers; they were asked to press “F” in response to any odd number (1,3,5,7,9) and “J” in response to any even number (2,4,6,8). If the word color was red, participants needed to decide whether the letter was a vowel or consonant; they were asked to press “F” in response to any vowels (A, E, I, O, U) and “J” in response to any consonants (G, K, M, R, etc.). After learning the rules in the practice phase, the participants entered the formal experiment. At the end of Experiment 3, data processing and analysis were performed on the total conversion response time of the shifting number task.

The Stroop task is used to examine individuals’ inhibition ability (Zhang et al., 2020). The task involved presenting a single-color word at the center of the screen; in the current experiment, one Chinese character was presented at a time, which was red, green, yellow, or blue and meant “red,” “green,” “yellow,” or “blue.” Sometimes the character and its color were the same, for example, the character “red” was red, and sometimes the character and its color were not the same, for example, the character “red” was green; every trial contained consistencies and inconsistencies. Participants were inquired to ignore the word and give a key-press in response to the color. The colors corresponded to the keys one by one (“D” for red, “F” for green, “J” for yellow, and “K” for blue). One block in the emotional Stroop task comprised 30 stimuli (i.e., trials). During each trial, each Chinese character remained until the participant responded or 2,000 ms passed, and after a 1,500 ms fixation was presented, the next stimulus appeared. Ten practice trials and a 3-min formal test were designed. The program gave feedback on correct or incorrect responses after the participant pressed the button during the practice trials, and there was no feedback during the formal tests irrespective of whether the response was correct or incorrect. At the end of Experiment 3, data processing and analysis were conducted by subtracting the response time of the inconsistent Stroop test from the response time of the consistent Stroop test.

#### 4.1.3. Procedure

The procedure was the same as in Experiment 2, but there was an extra phase before the answer phase. The new phase was the EFs measurement phase, which measured three dimensions in random order: working memory was measured by a two-back task, shifting by a shifting number task, and inhibition by the Stroop task. Participants had at most 3 min to complete each task. Note that the problems presentation and the prototypes learning phases presented different problems and prototypes from Experiment 2; in the current experiment, 24 high-difficulty problems were selected as materials.

### 4.2. Results

SPSS 22.0 was used for statistical analysis; relative mediation analyses were performed using the mediation package. To investigate the relations among the levels of consciousness, three sub-functions of EFs, two rates of prototype heuristics, and descriptive statistics are summarized in Table 3. Among them, the three sub-functions (inhibition, shifting, and updating) scores were calculated by the reaction time of the correct response to the task, in milliseconds. The correlation between each variable was analyzed and is presented in Table 4.

**Table 3:** Descriptive statistics of variables (*n* = 86; M ± SD; the total EF score has been deleted).

Variables	Conscious condition	Low-distractor task condition	High-distractor task condition
Updating	660.75 (132.08)	526.09 (66.38)	642.04 (136.96)
Shifting	1492.63 (141.50)	1067.50 (114.74)	1232.07 (162.19)
Inhibition	1034.87 (165.07)	749.36 (85.78)	853.13 (127.94)
The prototype activation rate	0.89 (0.09)	0.99 (0.02)	0.93 (0.03)
The accuracy rate	0.46 (0.14)	0.67 (0.10)	0.59 (0.12)

**Table 4:** Correlation analysis between variables (the total EF score has been deleted).

Variables	1	2	3	4	5	6
1 Conscious level	1					
2 Updating	0.43^**^	1				
3 Shifting	0.76^**^	0.16	1			
4 Inhibition	0.67^**^	0.23^*^	0.39^*^	1		
5 The prototype activation rate	0.80^**^	0.51^**^	0.71^*^	0.77^**^	1	
6 The accuracy rate	0.59^**^	0.48^**^	0.50^**^	0.56^**^	0.74^**^	1

* indicates that the correlation is significant at the level of 0.05; ** indicates that the correlation is significant at the level of 0.01.

As can be seen from Table 4, there were significant positive correlations between all conditions. Hereafter, mediation analyses were performed. As independent variables are categorical variables, and intermediate variables and dependent variables were continuous variables in the current experiment, bootstrap relative mediation analysis was performed using the mediation package (Hayes and Preacher, 2014; Jie and Wen, 2017). The independent variable levels of consciousness were coded, with the conscious condition as the reference variable, the high-distractor task condition as dummy variable 1, and the low-distractor task condition as dummy variable 2. Bootstrap set random sampling to 5,000 times, with the prototype activation rate and the accuracy rate as dependent variables under a 95% confidence interval. The global mediation analysis and relative mediation analysis were conducted with the three sub-functions of EFs as three parallel mediation variables. The results were as follows.

The total effect of the global mediation analysis with the prototype activation rate as the dependent variable was significant [*F*_(4,81)_ = 43.5, *p* < 0.001], indicating that the two relative total effects are not 0. The total direct effect of the global mediation analysis with the prototype activation rate as the dependent variable was also significant [*F*_(7,78)_ = 114.95, *p* < 0.001] and indicated that the two relative direct effects are not 0. The total effect of the global mediation analysis with the accuracy rate as the dependent variable was significant [*F*_(4,81)_ = 11.96, *p* < 0.001], indicating that the two relative total effects are not 0. The total direct effect of the global mediation analysis with the accuracy rate as the dependent variable was also significant [*F*_(7,78)_ = 13.31, *p* < 0.001], indicating that the two relative direct effects are not 0. Therefore, further relative mediation analysis had to be conducted.

#### 4.2.1. The prototype activation rate as the dependent variable

**Figure 5:**
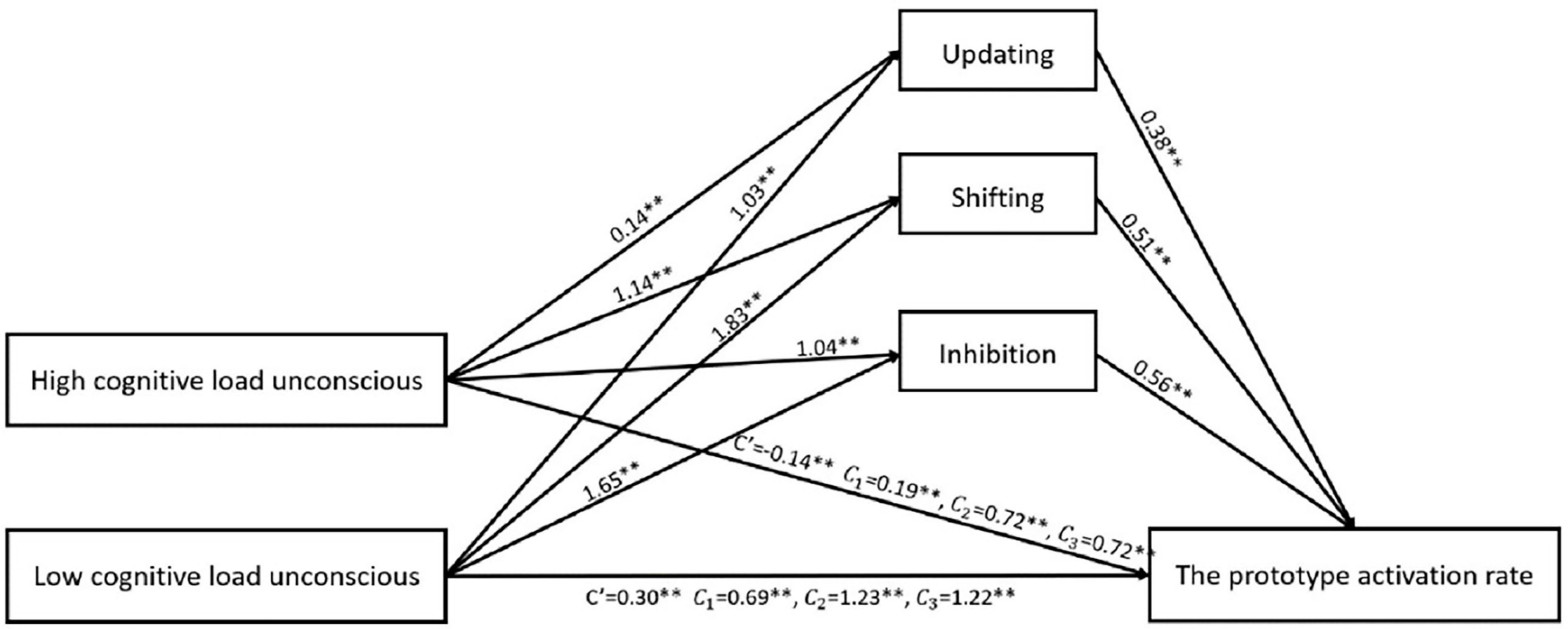
Mediation analysis with prototype activation rate as the dependent variable. ** indicates that the correlation is significant at the level of 0.01.

As shown in Figure 5, the relative mediation analysis, with the prototype activation rate as the dependent variable and levels of consciousness as the reference variable, showed that the working memory ability (updating) as the intermediate variable and the 95% bootstrap confidence interval between the high-distractor task and the conscious conditions was [0.15, 0.28], excluding 0, indicating significant relative mediation effect (a_11_ = 0.14, b_1_ = 0.38, a_11_b_1_ = 0.053). The 95% bootstrap confidence interval of the relative mediation analysis between the low-distractor task and conscious conditions was [0.19, 0.62], excluding 0, indicating a significant relative mediation effect (a_12_ = 1.03, b_1_ = 0.38, a_12_b_1_ = 0.39). These results suggest that high-and low-distractor tasks promote the updating ability of individuals and thus promote the ability of individuals to activate prototypes. However, the indirect mediating effect of the updating function was higher in the low-distractor task than in the high-distractor task condition.

With the prototype activation rate as the dependent variable, levels of consciousness as the reference variable, and shifting ability as the intermediate variable, the 95% bootstrap confidence interval between the high-distractor task and conscious conditions was [0.36, 0.79], excluding 0, indicating a significant relative mediation effect (a_21_ = 1.14, b_2_ = 0.51, a_21_b_2_ = 0.58); and the 95% bootstrap confidence interval between the low-distractor task and conscious conditions was [0.66, 1.17], excluding 0, indicating a significant relative mediation effect (a_22_ = 1.83, b_2_ = 0.51, a_22_b_2_ = 0.93). These results suggest that high-and low-distractor tasks promote the shifting ability of individuals and thus promote the ability of individuals to activate prototypes. However, the indirect mediating effect of the shifting function was higher in the low-distractor task than in the high-distractor task condition.

With the prototype activation rate as the dependent variable, levels of consciousness as the reference variable, and the inhibition ability as the intermediate variable, the 95% bootstrap confidence interval between the high cognitive load condition and the conscious condition was [0.26, 0.97], excluding 0, indicating a significant relative mediation effect (a_31_ = 1.04, b_3_ = 0.56, a_31_b_3_ = 0.58); and the 95% bootstrap confidence interval between the low-distractor task and conscious conditions was [0.51, 1.39], excluding 0, indicating a significant relative mediation effect (a_32_ = 1.65, b_3_ = 0.56, a_32_b_3_ = 0.92). These results suggest that high-and low-distractor tasks promote the inhibition ability of individuals, and thus promote the ability of individuals to activate prototypes. However, the indirect mediating effect of the inhibition function was higher in the low-distractor task than in the high-distractor task condition.

#### 4.2.2. The accuracy rate as the dependent variable

**Figure 6:**
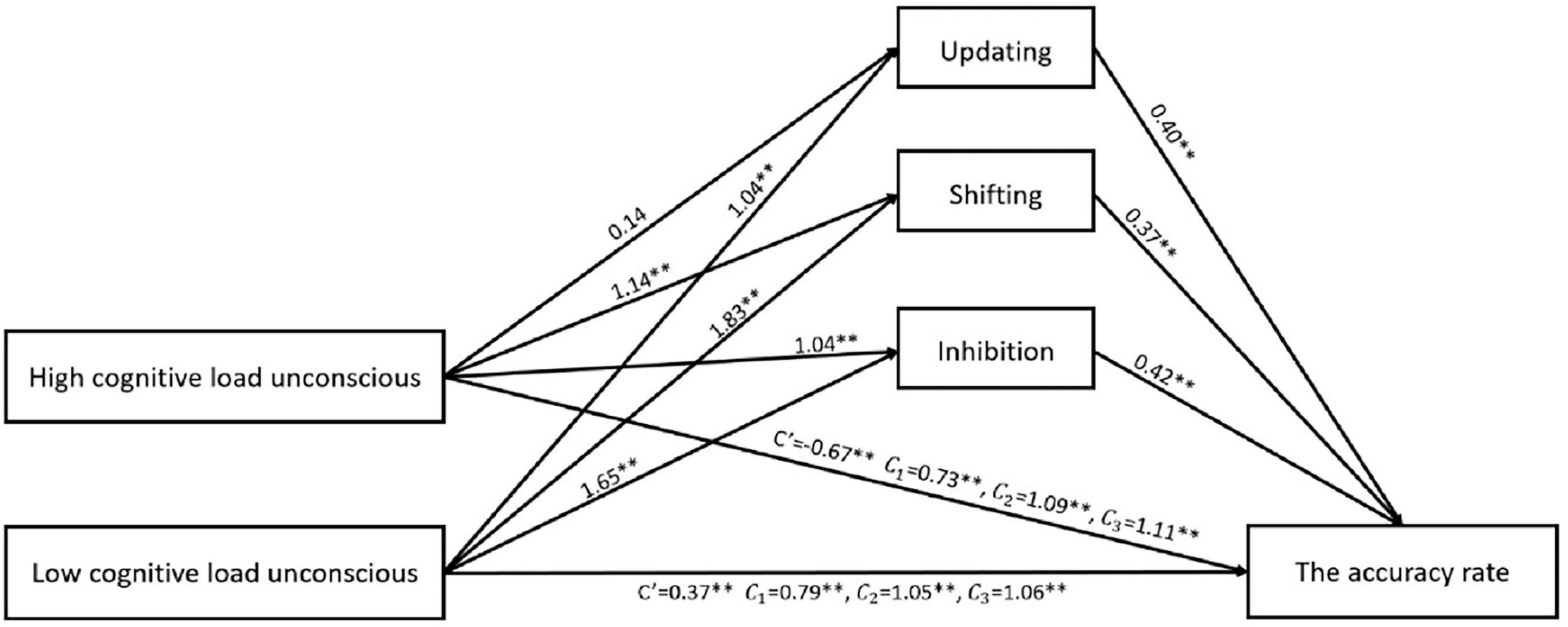
Mediation analysis with an accuracy rate as the dependent variable. ** indicates that the correlation is significant at the level of 0.01.

As shown in Figure 6, relative mediation analysis with the accuracy rate as the dependent variable, levels of consciousness as the reference variable, and working memory ability (updating) as the intermediate variable, showed that the 95% bootstrap confidence interval between the high-distractor task and conscious conditions was [−0.16, 0.33], including 0, indicating no significant relative mediation effect; and the 95% bootstrap confidence interval between the low-distractor task and conscious conditions was [0.18, 0.71], excluding 0, indicating a significant relative mediation effect (a_12_ = 1.04, b_1_ = 0.40, a_12_b_1_ = 0.42). These results suggest that, compared with the conscious condition, the high-distractor task condition does not promote the working memory ability of individuals, but low-distractor tasks promote individuals’ working memory ability and thus promote individuals’ ability to solve SIPs. In addition, the indirect mediating effect of the updating function was higher in the low-distractor task than in the high-distractor task condition.

The relative mediation analysis with the accuracy rate as the dependent variable, levels of consciousness as the reference variable, and the shifting ability as the intermediate variable showed that the 95% bootstrap confidence interval between the high-distractor task and conscious conditions was [0.09, 0.82], excluding 0, indicating a significant relative mediation effect (a_21_ = 1.14, b_2_ = 0.37, a_21_b_2_ = 0.42); and the 95% bootstrap confidence interval between the low-distractor task and conscious conditions was [0.15, 1.30], excluding 0, indicating a significant relative mediation effect (a_22_ = 1.83, b_2_ = 0.37, a_22_b_2_ = 0.68). These results suggest that, compared with the conscious condition, the high-and low-distractor task conditions promote the shifting ability of individuals, and thus promote individuals’ ability to solve SIPs. However, the indirect mediating effect of the shifting function was higher in the low-distractor task than in the high-distractor task condition.

The relative mediation analysis with the accuracy rate as the dependent variable, levels of consciousness as the reference variable, and the inhibition ability as the intermediate variable showed that the 95% bootstrap confidence interval between the high-distractor task and conscious conditions was [0.17, 0.76], excluding 0, indicating a significant relative mediation effect (a_31_ = 1.04, b_3_ = 0.42, a_31_b_3_ = 0.44); and the 95% bootstrap confidence interval between the low-distractor task and conscious conditions was [0.39, 1.10], excluding 0, indicating a significant relative mediation effect (a_32_ = 1.65, b_3_ = 0.42, a_32_b_3_ = 0.69). These results suggest that, compared with the conscious condition, the high-and low-distractor task conditions promote the inhibition ability of individuals, and thus promote individuals’ ability to solve SIPs. However, the indirect mediating effect of the inhibition function was higher in the low-distractor task than in the high-distractor task condition.

### 4.3. Discussion

The positive results of Experiment 3 supported our hypothesis and showed that, compared with the conscious condition, participants in the unconscious condition (low-distractor task and high-distractor task) had higher EFs. Moreover, participants who performed the low-distractor task also had higher EFs than participants who performed the high-distractor task, supporting the viewpoint that EFs can be depleted (Schmeichel, 2007). This means that previously conscious SIP solving occupies the largest amount of EFs resources, followed by the high-distractor task, and the low-distractor task occupies the least cognitive resources. Thus, conscious SIP solving occupies more cognitive resources than the distractor task. On the one hand, research has found that the correlation between insight and reasoning ability is as high as 0.920, but when the correlation between the two abilities is assumed to be 1, the model has a significant loss of fit, indicating that insight problem-solving and reasoning abilities highly overlap, although differently (Chuderski and Jastrzebski, 2018). Reasoning is an important ability that constitutes EFs (Chrysikou, 2019), so consciously solving insight problems will involve more EFs. On the other hand, Schmeichel (2007) also mentioned that distractor tasks (such as simple mathematical calculations) are achieved through automatic or regular cognitive processes that do not require a lot of EFs. Therefore, it is not surprising that SIP requires more EFs than distractor tasks.

Based on the above reasoning, after manipulating the level of consciousness, the rest of the EFs in the conscious condition was less than the high-distractor task and low-distractor task conditions, verifying that the EFs used in SIP in the conscious condition were less than the high-distractor task and low-distractor task conditions. The remaining EFs were positively correlated with the SIP-solving performance, which suggests that EFs contribute to unconscious SIP-solving. Specifically, when the prototype activation rate was used as the dependent variable, the three dimensions of EFs in the two distractor task groups had partial mediating effects compared with the control group, but the mediating effects of the three mediating variables in the high-distractor task condition were all smaller than those in the low-distractor task condition, which verified hypothesis b.

Of note, however, Korovkin et al. (2018) used the dual task to investigate the effect of different working memory systems’ load on insight problem-solving. They found that insight reorganization relies on fairly low levels of processing occurring in the working memory storage system, and the closer a person is to an insight solution, the more important the role of working memory in insight problem-solving becomes. This suggests that working memory is involved in insight problem-solving but at a very low level. Specifically, the difficulty of recalling memory content rather than the organization form affects an individual’s ability to make creative associations (Beaty et al., 2014). In the current experiment, the link between the prototype and the problem was already established at the unconscious level. To this end, bringing the connection to the conscious level requires very little updating ability, and the closer individuals get to the insight solution, the more important the role of updating working memory becomes. This reasoning also explains why updating has a significant mediating effect on the high and low cognitive load of prototype activation and a significant mediating effect on the low cognitive load of problem-solving, but not on the high cognitive load of problem-solving. This is because the prototype activation by working memory updating only needs to extract the key prototype, and the requirement of working memory updating is very small, but the problem solving of working memory updating needs to extract and problem solve the related characteristic of the prototype of the function and get the solution, which is more demanding on working memory updating. Therefore, working memory updating cannot be supported enough under a high cognitive load.

## 5. General discussion

The current study performed three experiments to investigate the difference in the effect of prototype heuristics in SIP solving with different levels of consciousness and explore its internal mechanism. The results found that after learning prototypes, distractor tasks induced unconscious processing, and when solving scientific innovation problems creatively by unconscious thinking, especially when the difficulty of the problem increased, the facilitation effect of unconscious processing became more prominent. The effect of unconscious processing was also related to the cognitive load of distractor tasks. This is generally consistent with previous studies on the relationship between unconscious processing and creative problems. Based on previous research, this research also studied the relationships among UC, EFs, and SIP and found that three dimensions of EFs (working memory, shifting, and inhibition) mediated the relationship between the level of consciousness and SIP solving. However, what is the specific process and mechanism of this action?

First, it is worth thinking about whether the executive function is a trait or an ability because different perspectives can lead to the opposite result. When we think of the executive function as a trait, the researchers will treat the measured executive function scores as a general level of executive control, and participants who have high executive function scores will have more resources to complete any task. However, if we think of the executive function as an ability, then the resource depletion hypothesis (Schmeichel, 2007) tells us that prior tasks deplete our executive control, and the executive function scores measured in later tasks was the amount of executive control ability that the participant has left available for this measuring task, the lower these scores, the higher the level of executive control the subject used in the previous task. In our experiment, the results of Experiment 3 can only be explained by taking the executive function as a kind of ability, that is, the differences in the executive function of different groups are caused by the differences in the operations that induce different levels of consciousness previously, rather than the differences in the pre-existing traits of different groups. Moreover, the order of such differences has been reasonably explained in the discussion of Experiment 3. Therefore, this study also provides additional support for the conclusion that executive function is an ability.

In addition, according to the prototype heuristic theory, the insight of SIP includes at least two stages: prototype activation and obtaining heuristics from a prototype. Prototype activation is automatic and obtaining heuristics requires executive control (Cao et al., 2006), which implies a cooperative mode of UC and EFs in SIP solving. Similarly, Beaty et al. (2016) summarized brain imaging research on creative thinking and found that many studies have pointed out the important role of default network and episodic memory in creative cognition; they suggested that the default network influences the generation of candidate ideas, while executive control guides and monitors them. The results of Experiment 3 supported this view, that is, compared to the high-distractor task condition, although the low-distractor task condition had a larger set of available EFs resources that led to better performance on SIP, participants who only performed conscious SIP solving had the worst performance, despite all their EFs resources used to solve the SIP. This means that although EFs are important for SIP solving, the result of problem-solving will be very poor if there is no unconscious processing, and EFs and UC are both indispensable in difficult insight problem-solving.

While many studies documented the positive role of EFs in creativity, others provide evidence to the contrary. For example, Chuderski and Jastrzebski (2018) concluded that to date, researchers’ studies on working memory and insight problem-solving have reported highly inconsistent results, ranging from moderately positive to zero and even negative effects. Zhu et al. (2019) discussed brain structure and resting brain function in SIP solving; they reported that decreased response inhibition, as well as the automatic association of semantics, will support representation-connection in the insight process. This suggests that the decrease in inhibition ability promotes semantic linkage during insight problem-solving, and thus facilitates problem-solving. We suggest that a paradoxical result of the different roles of EFs in insight problem-solving is that the UC and EFs resources required are different at different stages of insight problem-solving. That is, with the unconscious processing of the problem situation, key information retrieval, and the formation of semantic links, excessive EFs will hinder the process. Contrastingly, if the solution to the problem has already been found in the unconscious state, too little executive control will make individuals unable to extract the results to the level of consciousness and report them, thus affecting the performance of the subjects.

Past research has outlined this process. For example, the role of UC is to generate ideas by searching for materials in episodic memory (Beaty et al., 2016), or generate “structural mapping” and “representation-connection” between prototypes and problems (Zhu et al., 2019), while before, during, and after unconscious action, different levels of EFs play different roles. For example, many researchers believe that working memory is important in the early stage of insight problem-solving, such as problem understanding and goal orientation (Chrysikou, 2019), and in the later stage, Korovkin et al. (2018) suggested that working memory is more important the closer it is to problem-solving. Simple creativity tasks were not affected by working memory loads (Stuyck et al., 2022); it can be inferred that working memory also plays a role in extracting thoughts or links from the unconscious to the conscious level. In addition, inhibition plays an important role in suppressing conventional thoughts that are not novel when the individual is in cognitive fixation (Camarda et al., 2018), but also blocks UC-dominated representation-connection (Zhu et al., 2019). This suggests a complex relationship between the negative role of inhibitory control in unconscious processes and the important role it plays in the top-down overcoming of functional fixity. Lu et al. (2017) found that task switching can enhance creativity by reducing cognitive fixation, suggesting the role of switching in fixation, similar to inhibition. Ding et al. (2019) found that subjects’ performance in the Creative Scientific Problem Finding Test, regardless of the field, had no significant difference after conscious and unconscious thinking. However, in the Creative Scientific Problem Finding Test of a specific field, conscious thinking is superior to unconscious thinking, which may reveal that the role of conscious thinking is to screen and control creative thoughts in a specific situation so that creativity can better meet the direction required by the question.

**Figure 7:**
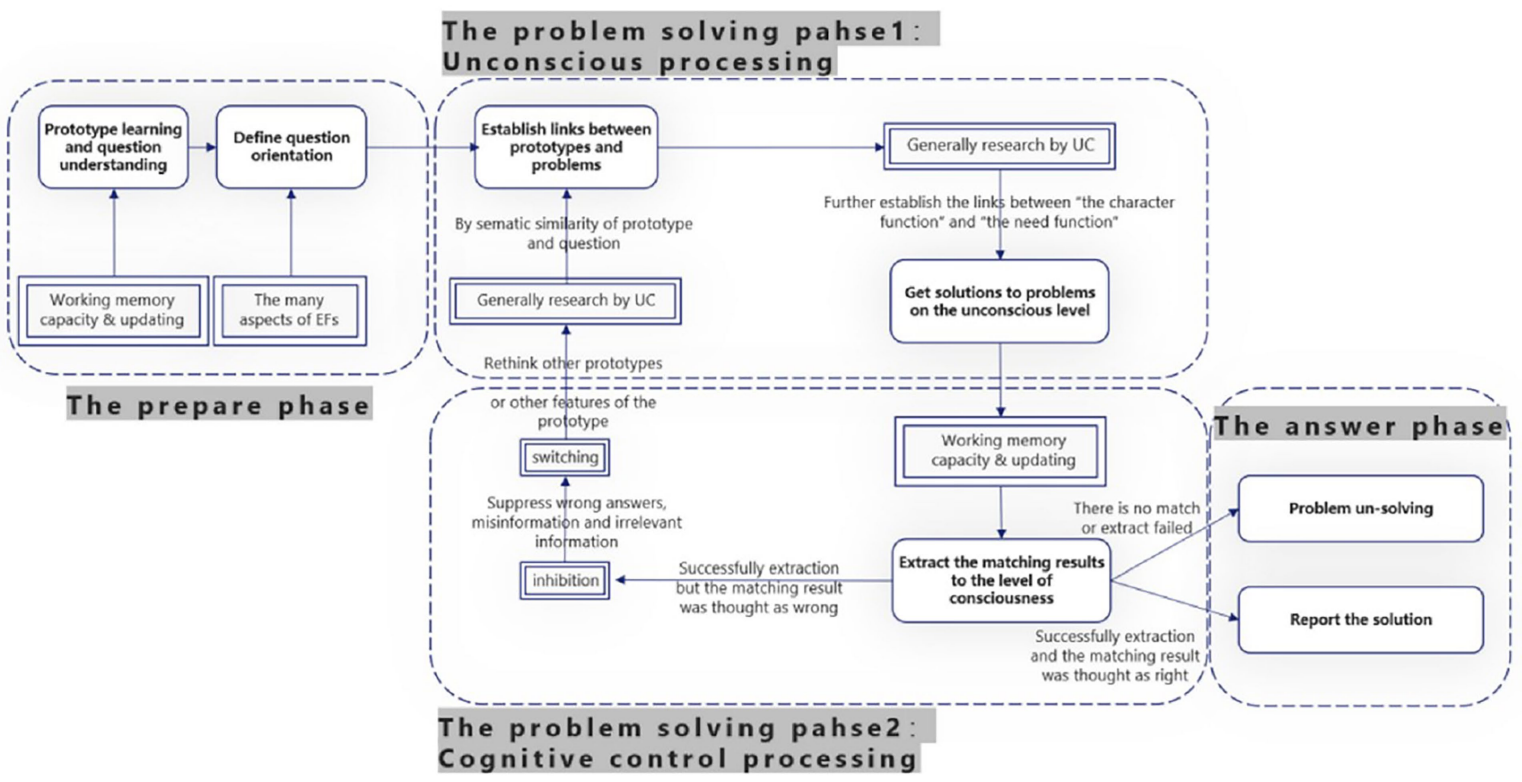
An insight prototype heuristic model of executive functions and unconsciousness. This model is only applicable to the problem-solving process of difficult insight problems with unconscious effects.

Finally, to further understand the role of UC and EFs in SIP solving, we propose a conjecture about this process based on the viewpoints of previous studies (see Figure 7). As can be seen from Figure 7, we divided the process of UC involving difficult insight problem-solving into three phases: prepared, problem-solving, and answer. Among them, the problem-solving phase was further divided into the first half dominated by UC and the second half dominated by EFs. In the preparation phase, working memory capacity and updating are used to learn and memorize insight problems (and prototype in our experiments), and EFs also help individuals form goal orientation. In the first half of the problem-solving phase, UC plays an important role that assesses its powerful search capabilities to retrieve questions and relevant prototypes and experiences, make new connections, and try to come up with answers. At this time, if EFs (such as inhibition) are too strong, it will cause certain damage to this part. In the second half of the problem-solving phase, working memory tries to extract related information to consciousness and to pick up the semantic links that had formed at the unconscious level; if the participants think they found the right solution, the method is reported (the answer phase), and if it is wrong, one can inhibit the wrong solution, suppress interference or irrelevant information, and use the ability to switch and overcome the fixation, think from a new perspective, and re-enter the cycle until a satisfactory answer is obtained and reported (the answer phase), or a satisfactory answer is not obtained and the problem is not solved (the answer phase).

Moreover, Yang et al. (2022) argued that it is unclear whether the state of creativity can have an impact on knowledge-rich creative problem-solving and whether interventions, that support analogical transfer in the heuristic prototype paradigm, can be used to improve knowledge-rich creative problem-solving. Current experimental manipulation and findings of this study provide definitive answers that, through certain distractor tasks, it is possible to improve knowledge-rich creative problem-solving, such as SIPs.

To summarize, we reported three experiments to explore the relationships among UC, EFs, and insight problem-solving, found that low cognitive load UC promotes prototype heuristics in SIPs, and proved more evidence for research in this area. To further understand the role of UC and EFs in SIP solving, we propose a conjecture about this process based on the viewpoints of previous studies.

### 5.1. Limitations

The current study first explored the relationships among UC, EFs, and insight problem-solving and proposed a new conjecture. However, direct evidence of the internal mechanism is somewhat insufficient, and future research can further verify the fuzzy zone. Second, this study uses SIPs as the insight problem, which can only show that the EFs and UC collaboration mode are such in solving the SIPs. The conclusion should be cautiously generalized, and future research can use other insight paradigms for more exploration.

## Data availability statement

The raw data supporting the conclusions of this article will be made available by the authors, without undue reservation.

## Ethics statement

The studies involving human participants were reviewed and approved by the Human Research Ethics Committee, Faculty of Psychology, Southwest University. The patients/participants provided their written informed consent to participate in this study.

## Author contributions

YL provided ideas for the argumentation and wrote the submitted manuscript. LT contributed to the conception and design of the study and wrote the first draft of the manuscript. LZ reviewed and edited the manuscript. GC contributed to study design and to manuscript drafting and reviewing. All authors contributed to the article and approved the submitted version.

## Funding

This study was supported by Southwest University open access funding and Subject on Social Science of Chongqing Medical and Pharmaceutical College (ygz2022203).

## Conflict of interest

The authors declare that the research was conducted in the absence of any commercial or financial relationships that could be construed as a potential conflict of interest.

## Publisher’s note

All claims expressed in this article are solely those of the authors and do not necessarily represent those of their affiliated organizations, or those of the publisher, the editors and the reviewers. Any product that may be evaluated in this article, or claim that may be made by its manufacturer, is not guaranteed or endorsed by the publisher.
